# Association of Long Noncoding RNA HOTAIR Polymorphism and the Clinical Manifestations of Diabetic Retinopathy

**DOI:** 10.3390/ijerph192114592

**Published:** 2022-11-07

**Authors:** Chih-Chun Chuang, Kai Wang, Yi-Sun Yang, Edy Kornelius, Chih-Hsin Tang, Chia-Yi Lee, Hsiang-Wen Chien, Shun-Fa Yang

**Affiliations:** 1 Institute of Medicine, Chung Shan Medical University, Taichung 402, Taiwan; 2 Department of Ophthalmology, Changhua Christian Hospital, Changhua 500, Taiwan; 3 Department of Post-Baccalaureate Medicine, College of Medicine, National Chung Hsing University, Taichung 402, Taiwan; 4 Department of Ophthalmology, Cathay General Hospital, Taipei 106, Taiwan; 5 Departments of Ophthalmology, Sijhih Cathay General Hospital, New Taipei City 221, Taiwan; 6 School of Medicine, College of Medicine, Fu Jen Catholic University, New Taipei City 242, Taiwan; 7 School of Medicine, Chung Shan Medical University, Taichung 402, Taiwan; 8 Department of Internal Medicine, Division of Endocrinology and Metabolism, Chung Shan Medical University Hospital, Taichung 402, Taiwan; 9 School of Medicine, China Medical University, Taichung 404, Taiwan; 10 Department of Medical Laboratory Science and Biotechnology, College of Medical and Health Science, Asia University, Taichung 413, Taiwan; 11 Department of Ophthalmology, Nobel Eye Institute, Taipei 115, Taiwan; 12 School of Medicine, National Tsing Hua University, Hsinchu 300, Taiwan; 13 Department of Medical Research, Chung Shan Medical University Hospital, Taichung 402, Taiwan

**Keywords:** lung cancer, GAS5, polymorphism, EGFR mutation

## Abstract

The aim of the current study is to evaluate the possible correlation between the single-nucleotide polymorphisms (SNP) of HOX transcript antisense intergenic RNA (HOTAIR) and the clinical characteristics of diabetic retinopathy (DR). Four loci of HOTAIR SNPs, including rs920778 (T/C), rs12427129 (C/T), rs4759314 (A/G), and rs1899663 (G/T), were genotyped via the TaqMan allelic discrimination for 276 DR individuals and 452 non-DR patients. The distribution frequency of HOTAIR SNP rs12427129 CT [adjusted odds ratio (AOR): 1.571, 95% CI: 1.025–2.408, *p* = 0.038], HOTAIR SNP rs12427129 CT+TT (AOR: 1.611, 95% CI: 1.061–2.446, *p* = 0.025), and HOTAIR SNP rs1899663 TT (AOR: 2.443, 95% CI: 1.066–5.595, *p* = 0.035) were significantly higher in the DR group. Moreover, the proliferative diabetic retinopathy (PDR) subgroup revealed a significantly higher distribution of HOTAIR SNP rs12427129 CT+TT (AOR: 2.016, 95% CI: 1.096–3.710, *p* = 0.024) and HOTAIR SNP rs1899663 TT (AOR: 4.693, 95% CI: 1.765–12.479, *p* = 0.002), and the distribution frequencies of HOTAIR SNP rs12427129 CT (AOR: 3.722, 95% CI: 1.555–8.909, *p* = 0.003), HOTAIR SNP rs12427129 CT+TT (AOR: 4.070, 95% CI: 1.725–9.600, *p* = 0.001), and HOTAIR SNP rs1899663 TT (AOR: 11.131, 95% CI: 1.521–81.490, *p* = 0.018) were significantly higher in the female PDR subgroup. Regarding the clinical characters, the DR patients with HOTAIR SNP rs1899663 GT+TT revealed a significantly shorter duration of diabetes compared to the DR patients with HOTAIR SNP rs1899663 GG (10.54 ± 8.19 versus 12.79 ± 7.73, *p* = 0.024). In conclusion, HOTAIR SNP rs12427129 and rs1899663 are strongly correlated to the presence of DR, especially for a female with PDR.

## 1. Introduction

Diabetic retinopathy (DR) is a prevalent microvascular complication of diabetes; about one-third of diabetes patients develop this morbidity [1,2]. DR can be further divided into non-proliferative diabetic retinopathy (NPDR) and proliferative diabetic retinopathy (PDR) depending on the existence of neovascularization [3]. The presence of PDR can contribute to severe visual impairments; DR is the cause of 1.1% of all blindness cases globally [4]. Managing DR includes lifestyle modifications and blood sugar control; pan-retinal photocoagulation, intravitreal injection of the anti-vascular endothelial growth factor, or steroids would be applied in vision-threatening DR [5].

Certain factors have been established as predisposing factors for DR [6]. Hyperglycemia, higher hemoglobin A1C (HbA1c) levels, and hypertension are by far the most important risk factors for DR development [7,8]. In previous studies, the risk of DR elevated 1.15-fold in individuals with 1% increments of HbA1c [6]. Moreover, advanced glycation end products and higher serum lipid levels, including high LDL and triglycerides, are related to DR [9]. In addition, genetic polymorphism can increase one’s chances of developing DR. Known genetic risk factors for DR include the single-nucleotide polymorphism (SNP) of growth arrest-specific 5, according to a previous publication [10].

HOX transcript antisense intergenic RNA (HOTAIR) is a type of long noncoding RNA that regulates the chromatin dynamics and associates with various types of cancers [11,12]. According to previous research studies, the malignancies associated with the polymorphism of HOTAIR include lung cancer, oral cancer, and urothelial cell carcinoma [13,14,15]. Moreover, HOTAIR could promote hypoxia and the subsequent ischemic reaction [16]. Regarding the association between HOTAIR and metabolic disorders or related co-morbidities, low expressions of HOTAIR in peripheral blood lymphocytes were observed in patients with atherosclerosis [17]. In another study, the high level of HOTAIR was induced by hyperglycemia and promoted retinal angiogenesis; increased HOTAIR expression was found in the vitreous humor of patients with proliferative diabetic retinopathy [18]. Nevertheless, there was scant evidence regarding the correlation between HOTAIR SNP and DR. Because HOTAIR could regulate the ischemic process, which is a major pathophysiology of DR [5,16], the association warrants additional validation.

The purpose of our study was to evaluate the potential relationship between the genetic polymorphism of HOTAIR and the clinical characteristics of DR. The patients with NPDR and PDR were analyzed separately in the subgroup analyses.

## 2. Materials and Methods

### 2.1. Subject Selection

We conducted a prospective case-control study at Chung Shan Medical University Hospital, which is a tertiary hospital in the central Taiwan region. After explaining the details of our study, a total of 728 patients with diabetes participated. We reviewed the medical documents from the ophthalmic department; 276 and 452 participants were categorized into the DR and non-DR groups. Moreover, 165 and 111 participants with DR were categorized into NPDR and PDR subgroups. DR in our study was regarded as one of the following retinal defects: hard exudate, dot/flame-shaped hemorrhage, microaneurysm, cotton–wool spots, venous beading, or intraretinal microvascular abnormalities. PDR was defined as the development of one of the following retinal disorders: neovascularization of the optic disc or retina, vitreous hemorrhage, tractional retinal detachment, or neovascular glaucoma. This study was approved by the Institutional Review Boards of Chung Shan Medical University Hospital (project identification code: CS1-20048). In addition, informed consent was signed by each participant in the current study.

### 2.2. Medical Information and Samples Acquirement

Medical data involving age, sex, diabetes duration, lipid profiles, HbA1c, renal function, and the injection of insulin were obtained. For the approaches of HOTAIR genes and related polymorphism, venous blood extraction was executed in all patients; the blood samples were then stored in the ethylenediaminetetraacetic acid-containing tubes. Immediately, the samples were centrifuged and restored in a laboratory refrigerator at approximately −80 °C. Individuals were removed from our study if their genomes in the samples were corrupted before our analyses.

### 2.3. DNA Obtain and Definition of HOTAIR SNP with Real-Time PCR

Four HOTAIR SNPs, rs920778 (T/C), rs12427129 (C/T), rs4759314 (A/G), and rs1899663 (G/T), were selected for analyses because the earlier studies demonstrated the association between these SNPs and malignancy or metabolic disorders [14,15,19,20]. The DNA collection and analysis methods in the current study refer to procedures from our previous publication [21,22]. We extracted genomes and DNA from leukocytes in blood samples via QIAamp DNA kits (Qiagen, Valencia, Valencia, CA, USA) according to the manufacturer’s instructions concerning DNA isolation. The isolated DNA was restored in a refrigerator at approximately −20 °C. The polymorphisms of HOTAIR SNPs rs920778 (T/C), rs12427129 (C/T), rs4759314 (A/G), and rs1899663 (G/T) were sequenced via the ABI StepOne Real-Time PCR System (Applied Biosystems, Foster City, CA, USA); these HOTAIR polymorphism findings were further analyzed and enhanced by SDS version 3.0 software (Applied Biosystems). The primers used for the HOTAIR genotype are listed in Table S1. The representative results of the TaqMan assay for four HOTAIR SNPs are shown in Figures S1–S4.

### 2.4. Statistical Analysis

SAS version 9.4 (SAS Institute, Inc., Cary, NC, USA) was utilized for the analyses in the current study. We used the mean value with the standard deviation (SD) or the percentage to illustrate the basic characteristics between the two groups, depending on the nature of the parameters. Moreover, we used an independent t test to measure the differences in the basic characteristics between the non-DR and DR groups. After that, we utilized the multiple logistic regression models to yield the adjusted odds ratios (AORs) with the corresponding 95% confidence intervals (CI) of the four SNP distributions between the two groups. The multiple logistic regression models controlled the effects of age, HbA1c, serum creatinine levels, insulin treatment, duration of diabetes, glomerular filtration rate, and HDL cholesterol levels. The same multiple logistic regression models were employed in the subgroup analyses concerning the four SNP distributions between the NPDR and PDR subgroups and the non-DR population. Similarly, multiple logistic regression models were used to compare the four SNP distributions between the PDR and non-DR women. Furthermore, the differences in diabetes duration in the SNP rs1899663 wild type (GG) and the variant (GT+TT) in diabetes patients with different DR stages were analyzed via the independent *t* test. A *p* value lower than 0.05 is defined as statistically significant.

## 3. Results

### 3.1. Clinical Manifestation between the Non-DR and DR Populations

The clinical and laboratory characteristics of the non-DR and DR groups are shown in Table 1. The mean age was 62.61 ± 10.72 years old in the DR group, which was significantly older than the 60.24 ± 11.22 years old in the non-DR group (*p* = 0.005). In addition, the DR population revealed a longer duration of diabetes, higher HbA1c levels, a higher rate of insulin treatment, higher serum creatinine, a lower glomerular filtration rate, and lower HDL cholesterol levels compared to the non-DR group (all *p* < 0.05) (Table 1).

### 3.2. The Distribution Frequencies of HOTAIR SNP among Different Diabetes Populations

In the comparison between the DR and non-DR group regarding HOTAIR SNPs, the three genotypic frequencies of HOTAIR SNPs in the non-DR group conformed to the Hardy–Weinberg equilibrium (rs12427129: *p* = 0.582; rs4759314: *p* = 0.315 and rs1899663: *p* = 0.080). Moreover, the distribution frequencies of HOTAIR SNP rs12427129 CT (AOR: 1.571, 95% CI: 1.025–2.408, *p* = 0.038), HOTAIR SNP rs12427129 CT+TT (AOR: 1.611, 95% CI: 1.061–2.446, *p* = 0.025), and HOTAIR SNP rs1899663 TT (AOR: 2.443, 95% CI: 1.066–5.595, *p* = 0.035) were significantly higher in the DR group compared to the wild type (Table 2). However, the distribution frequencies of all HOTAIR SNPs between the DR and NPDR populations were similar compared to the wild type (Table 3). Regarding the distribution frequency between the DR and PDR populations, the PDR subgroup revealed a significantly higher distribution of HOTAIR SNP rs12427129 CT+TT (AOR: 2.016, 95% CI: 1.096–3.710, *p* = 0.024) and HOTAIR SNP rs1899663 TT (AOR: 4.693, 95% CI: 1.765–12.479, *p* = 0.002) compared to the wild type (Table 4). Moreover, the distribution frequencies of HOTAIR SNP rs12427129 CT (AOR: 3.722, 95% CI: 1.555–8.909, *p* = 0.003), HOTAIR SNP rs12427129 CT+TT (AOR: 4.070, 95% CI: 1.725–9.600, *p* = 0.001), and HOTAIR SNP rs1899663 TT (AOR: 11.131, 95% CI: 1.521–81.490, *p* = 0.018) were significantly higher in the PDR subgroup compared to the wile type when considering the female non-DR and PDR patients only (Table 5).

### 3.3. The Duration of Diabetes and the Genotypes of HOTAIR SNP rs1899663 in Diabetes Patients

Concerning the duration of diabetes based on the HOTAIR SNP rs1899663 variant, the DR patients with HOTAIR SNP rs1899663 GT+TT illustrated a significantly shorter duration of diabetes compared to the DR patients with HOTAIR SNP rs1899663 GG (10.54 ± 8.19 versus 12.79 ± 7.73, *p* = 0.024). Nevertheless, the durations of diabetes in different HOTAIR SNPs rs1899663 showed insignificant differences throughout the whole diabetes population, non-DR group, NPDR subgroup, and PDR subgroup (Table 6).

## 4. Discussion

In the current study, the DR group demonstrated a significantly higher distribution frequency of HOTAIR SNP rs12427129 CT, HOTAIR SNP rs12427129 CT+TT, and HOTAIR SNP rs1899663 TT. Moreover, female PDR patients showed a higher distribution frequency in the three above-mention HOTAIR SNPs. Moreover, the duration of diabetes was significantly shorter in the DR population with the HOTAIR SNP rs1899663 GT+TT variant compared to the DR population with wild type.

The HOTAIR gene has a major effect on cellular homeostasis and plays a critical role in many types of cancers [19,23,24,25,26]. The high level of HOTAIR expression is related to the development, proliferation, and metastasis of lung cancer [27]. Moreover, the HOTAIR level acts as an oncogene in patients with gastric cancer [26]. A previous study showed that HOTAIR is strongly involved in the initiation, metastasis, and drug resistance of breast cancer [28]. In addition to the HOTAIR level, the genetic polymorphism of HOTAIR can alter the development or progression of neoplasm. In a previous study, HOTAIR SNP rs1899663 was correlated to the risk of oral squamous cell carcinoma development after adjusting for several risk factors [15]. Urothelial cell carcinoma development and a lower overall survival rate are associated with HOTAIR SNP rs4759314 AG+GG [14]. Moreover, HOTAIR SNP rs1899663 was a risk factor for lung cancer in a meta-analysis [13]. In addition to malignancy, HOTAIR and its SNP can affect the clinical courses of vascular disorders in which HOTAIR can induce the ischemic infarct response via the upregulation of the NOX2 pathway [16]. Another study also showed that HOTAIR can increase oxidative stress and cardiac myocyte apoptosis [29]. In recent research, HOTAIR was a crucial mediator in an acute myocardial infarction event [30]. On the other hand, DR also has several genetic predisposing factors, such as growth arrest-specific 5 and long noncoding RNA LINC00673 [10,31]. SNP rs1990145 on chromosome 2 is highly associated with diabetic macular edema, and SNP rs918519 on chromosome 5 showed a significant presence in individuals with PDR [32]. Moreover, the osteoprotegerin SNP rs3134069 is correlated to the higher incidence of DR [33], and the arginase 1 SNP rs2781666 has a significantly higher susceptibility of DR in the type-2 diabetes population [34]. On the contrary, the polymorphisms of vascular endothelial growth factors, including rs3025035, rs3025021, and rs2010963, do not significantly alter the probability of PDR [35]. In the family of long non-coding RNAs, the long non-coding RNA MEG3 can reduce retinal neovascularization and prevent retinal injury from hyperglycemia [36]. However, the long non-coding RNA MALAT1, MIAT, and HOTAIR could enhance the progression of retinal neovascularization [36], which indicates that long non-coding RNAs have various and prominent effects on the retinal vasculature. Regarding the pathophysiology of DR—oxidative stress, ischemic response, and inflammation are the main ingredients [3,37,38]. Moreover, current evidence demonstrates that the existence of HOTAIR can affect the hypoxia, ischemia, and angiogenesis pathways in several tissues, including the retina [16,30,36]. Consequently, we suppose that the SNP of HOTAIR may also influence these pathways and is related to the development of DR at different stages. Our hypothesis is supported by the findings of the current study to some degree.

The current study shows that the distribution frequencies of HOTAIR SNP rs12427129 CT, HOTAIR SNP rs12427129 CT+TT, and HOTAIR SNP rs1899663 TT are significantly higher in the DR patients than the non-DR patients. To our knowledge, this was a preliminary experience that showed the potential correlation between DR and the SNP of HOTAIR. Furthermore, we adjusted several known risk factors for DR in the multivariable analysis, including age, HbA1c, serum lipid, and duration of diabetes. Consequently, the relationship between DR and related HOTAIR SNP should be strong. In a previous study, HOTAIR significantly promoted angiogenesis via STAT3-mediated inflammation [39]. Although no HOTAIR SNP was proven to alter ischemic or oxidative stress, HOTAIR SNP rs1899663 can alter psoriasis, which is an inflammatory disease [40]. Since an inflammation mechanism is a pathophysiology of DR [5], the distribution frequencies of specific HOTAIR SNPs could be higher in DR patients. However, further research is needed to clarify this concept. The distribution frequencies of the HOTAIR SNP rs920778 variant and HOTAIR SNP rs4759314 variant showed insignificant differences between the DR and non-DR groups in all of the analyses, which may imply minimal effects of the two HOTAIR SNPs on the clinical course of DR.

In the subgroup analyses, HOTAIR SNP rs12427129 CT+TT and HOTAIR SNP rs1899663 TT revealed higher distribution frequencies in the PDR population compared to the non-DR group. PDR is a severe DR status that presents with neovascularization, vitreous hemorrhage, tractional retinal detachment, and neovascular glaucoma [1]. In advanced PDR, vision loss could be prominent and even irreversible [41]. In the field of biochemistry, the higher vascular endothelial growth factor and inflammatory cells have been found in the retina with PDR [42,43]. The higher distribution frequency of HOTAIR SNP rs12427129 CT+TT and HOTAIR SNP rs1899663 TT in PDR patients may indicate that ischemic and inflammatory pathways may be correlated to the HOTAIR polymorphism due to the higher expression of the HOTAIR SNP variant in these patients. The non-significant differences in HOTAIR SNP distributions between the non-DR group and NPDR subgroup further strengthen this concept: the change of the HOTAIR polymorphism only presents in PDR individuals with severe ischemic and inflammatory responses. On the other hand, females with PDR demonstrated significantly higher distribution frequencies of HOTAIR SNP rs12427129 CT, HOTAIR SNP rs12427129 CT+TT, and HOTAIR SNP rs1899663 TT than females without DR, compared to the HOTAIR wild types. The significance of HOTAIR SNPs distribution frequencies in the female PDR population was the same throughout the entire DR population in comparison to the entire PDR population. This finding may indicate that female patients with DR are more susceptible to the genetic polymorphism of HOTAIR.

Regarding the clinical course of DR, individuals with both the DR and HOTAIR SNP rs1899663 GT+TT variant revealed significantly shorter durations of diabetes. Since the duration of diabetes is a prominent risk factor for the development of DR and a shorter duration of diabetes implies that DR occurs earlier after the presence of diabetes [6,7], the HOTAIR SNP rs1899663 GT+TT variant may aggregate the clinical course of DR. Indeed, the HOTAIR SNP rs1899663 TT variant showed a higher distribution frequency in both individuals with DR and PDR, which we discussed in a previous section. Moreover, although not significant, NPDR and PDR with the HOTAIR SNP rs1899663 GT+TT variant illustrated diabetes duration that was approximately two years shorter than DR patients without the HOTAIR polymorphism. Accordingly, HOTAIR SNP rs1899663 may be a prominent genetic factor in the development and course of DR compared to the other polymorphism of HOTAIR.

## 5. Conclusions

In conclusion, the distribution frequencies of HOTAIR SNP rs12427129 CT, HOTAIR SNP rs12427129 CT+TT, and HOTAIR SNP rs1899663 TT were significantly higher in DR patients, especially females with PDR. Furthermore, DR participants with HOTAIR SNP rs1899663 GT+TT showed significantly shorter durations of diabetes. Consequently, the genetic survey of HOTAIR polymorphism may be recommended for DR individuals with rapid progression. Further, large-scale prospective studies to evaluate the correlation between the HOTAIR polymorphism and the therapeutic outcome of DR are mandatory.

## Figures and Tables

**Table 1:** Clinical and laboratory characteristics of patients with diabetic retinopathy and non-diabetic retinopathy.

Variable	Non-DR Group (n = 452)	DR Group (n = 276)	*p* Value
Age (years)	60.24 ± 11.22	62.61 ± 10.72	0.005 *
Male gender [n (%)]	238 (52.7%)	152 (55.1%)	0.526
Duration of diabetes (years)	9.40 ± 7.04	11.98 ± 7.96	<0.001 *
HbA1c (% (mmol/mol))	6.96 ± 0.99	7.59 ± 1.42	<0.001 *
Insulin treatment (n (%))	105 (23.2%)	129 (46.7%)	<0.001 *
Serum creatinine (mg/dL)	0.89 ± 0.35	1.55 ± 1.84	<0.001 *
Glomerular filtration rate (mL/min)	78.41 ± 27.74	62.88 ± 34.15	<0.001 *
Total cholesterol (mmol/L)	160.65 ± 43.04	165.27 ± 47.61	0.186
HDL cholesterol (μmol/L)	46.35 ± 12.63	43.87 ± 13.58	0.014 *
LDL cholesterol (μmol/L)	86.66 ± 28.23	86.35 ± 32.86	0.894
Triglycerides, (μmol/L)	140.29 ± 165.28	156.80 ± 117.95	0.157

DR: diabetic retinopathy, n: number, * denotes significant differences between groups.

**Table 2:** Odds ratio and 95% confidence interval of diabetic retinopathy associated with HOTAIR genotypic frequencies.

Variable	Non-DR Group (n = 452)	DR Group (n = 276)	AOR (95% CI)	*p* Value
rs920778				
TT	210 (46.5%)	141 (51.1%)	1.000 (reference)	
TC	209 (46.2%)	113 (40.9%)	0.904 (0.633–1.290)	0.577
CC	33 (7.3%)	22 (8.0%)	1.146 (0.605–2.172)	0.676
TC+CC	242 (53.5%)	135 (48.9%)	0.938 (0.668–1.318)	0.714
rs12427129				
CC	370 (81.9%)	212 (76.8%)	1.000 (reference)	
CT	79 (17.5%)	61 (22.1%)	1.571 (1.025–2.408)	0.038 *
TT	3 (0.6%)	3 (1.1%)	2.567 (0.490–13.435)	0.264
CT+TT	82 (18.1%)	64 (23.2%)	1.611 (1.061–2.446)	0.025 *
rs4759314				
AA	364 (80.5%)	235 (85.1%)	1.000 (reference)	
AG	81 (18.0%)	40 (14.5%)	0.783 (0.494–1.242)	0.298
GG	7 (1.5%)	1 (0.4%)	0.205 (0.022–1.894)	0.163
AG+GG	88 (19.5%)	41 (14.9%)	0.733 (0.466–1.154)	0.180
rs1899663				
GG	284 (62.8%)	177 (64.1%)	1.000 (reference)	
GT	156 (34.5%)	83 (30.1%)	0.961 (0.661–1.397)	0.836
TT	12 (2.7%)	16 (5.8%)	2.443 (1.066–5.595)	0.035 *
GT+TT	168 (37.2%)	99 (35.9%)	1.081 (0.758–1.542)	0.666

DR: diabetic retinopathy, n: number, CI: confidence intervals, AOR: adjusted odds ratio, with their 95% confidence intervals were estimated by multiple logistic regression models after controlling for age, the duration of diabetes, HbA1c, insulin treatment, serum creatinine levels, glomerular filtration rate, and HDL cholesterol levels. * Denotes significant differences between groups.

**Table 3:** Odds ratio and 95% confidence interval of non-proliferative diabetic retinopathy associated with HOTAIR genotypic frequencies.

Variable	Non-DR Group (n = 452)	NPDR Subgroup (n = 165)	AOR (95% CI)	*p* Value
rs920778				
TT	210 (46.5%)	83 (50.3%)	1.000 (reference)	
TC	209 (46.2%)	73 (44.2%)	0.957 (0.641–1.430)	0.831
CC	33 (7.3%)	9 (5.5%)	0.637 (0.268–1.515)	0.308
TC+CC	242 (53.5%)	82 (49.7%)	0.911 (0.618–1.344)	0.639
rs12427129				
CC	370 (81.9%)	129 (78.2%)	1.000 (reference)	
CT	79 (17.5%)	35 (21.2%)	1.449 (0.890–2.360)	0.136
TT	3 (0.6%)	1 (0.6%)	1.238 (0.123–12.495)	0.857
CT+TT	82 (18.1%)	36 (21.8%)	1.441 (0.891–2.329)	0.136
rs4759314				
AA	364 (80.5%)	141 (85.5%)	1.000 (reference)	
AG	81 (18.0%)	24 (14.5%)	0.739 (0.431–1.266)	0.271
GG	7 (1.5%)	0 (0.0%)	–––	–––
AG+GG	88 (19.5%)	24 (14.5%)	0.676 (0.396–1.155)	0.152
rs1899663				
GG	284 (62.8%)	105 (63.6%)	1.000 (reference)	
GT	156 (34.5%)	53 (32.1%)	1.015 (0.667–1.544)	0.945
TT	12 (2.7%)	7 (4.3%)	1.472 (0.512–4.229)	0.473
GT+TT	168 (37.2%)	60 (36.4%)	1.051 (0.701–1.576)	0.810

DR: diabetic retinopathy, n: number, NPDR: non-proliferative diabetic retinopathy, CI: confidence intervals, AOR: adjusted odds ratio, with their 95% confidence intervals were estimated by multiple logistic regression models after controlling for age, the duration of diabetes, HbA1c, insulin treatment, serum creatinine levels, glomerular filtration rate, and HDL cholesterol levels.

**Table 4:** Odds ratio and 95% confidence interval of proliferative diabetic retinopathy associated with HOTAIR genotypic frequencies.

Variable	Non-DR Group (n = 452)	PDR Subgroup (n = 111)	AOR (95% CI)	*p* Value
rs920778				
TT	210 (46.5%)	58 (52.3%)	1.000 (reference)	
TC	209 (46.2%)	40 (36.0%)	0.736 (0.431–1.258)	0.263
CC	33 (7.3%)	13 (11.7%)	2.074 (0.948–4.537)	0.068
TC+CC	242 (53.5%)	53 (47.7%)	0.993 (0.600–1.641)	0.977
rs12427129				
CC	370 (81.9%)	83 (74.8%)	1.000 (reference)	
CT	79 (17.5%)	26 (23.4%)	1.869 (0.993–3.518)	0.052
TT	3 (0.6%)	2 (1.8%)	5.177 (0.799–33.549)	0.085
CT+TT	82 (18.1%)	28 (25.2%)	2.016 (1.096–3.710)	0.024 *
rs4759314				
AA	364 (80.5%)	94 (84.7%)	1.000 (reference)	
AG	81 (18.0%)	16 (14.4%)	0.905 (0.462–1.772)	0.770
GG	7 (1.5%)	1 (0.9%)	0.760 (0.082–7.073)	0.809
AG+GG	88 (19.5%)	17 (15.3%)	0.893 (0.465–1.713)	0.733
rs1899663				
GG	284 (62.8%)	72 (64.9%)	1.000 (reference)	
GT	156 (34.5%)	30 (27.0%)	0.795 (0.439–1.438)	0.447
TT	12 (2.7%)	9 (8.1%)	4.693 (1.765–12.479)	0.002 *
GT+TT	168 (37.2%)	39 (35.1%)	1.102 (0.649–1.872)	0.720

DR: diabetic retinopathy, n: number, PDR: proliferative diabetic retinopathy, CI: confidence intervals, AOR: adjusted odds ratio, with their 95% confidence intervals were estimated by multiple logistic regression models after controlling for age, the duration of diabetes, HbA1c, insulin treatment, serum creatinine levels, glomerular filtration rate, and HDL cholesterol levels. * Denotes significant differences between groups.

**Table 5:** Odds ratio and 95% confidence interval of proliferative diabetic retinopathy associated with HOTAIR genotypic frequencies in the female group.

Variable	Non-DR Group (n = 214)	PDR Subgroup (n = 57)	AOR (95% CI)	*p* Value
rs920778				
TT	101 (47.2%)	31 (54.4%)	1.000 (reference)	
TC	101 (47.2%)	21 (36.8%)	0.724 (0.338–1.547)	0.404
CC	12 (5.6%)	5 (8.8%)	1.377 (0.393–4.829)	0.617
TC+CC	113 (52.8%)	26 (45.6%)	0.816 (0.401–1.659)	0.574
rs12427129				
CC	182 (85.0%)	40 (70.2%)	1.000 (reference)	
CT	32 (15.0%)	16 (28.1%)	3.722 (1.555–8.909)	0.003 *
TT	0 (0.0%)	1 (1.7%)	–––	–––
CT+TT	32 (15.0%)	17 (29.8%)	4.070 (1.725–9.600)	0.001 *
rs4759314				
AA	173 (80.8%)	47 (82.5%)	1.000 (reference)	
AG	36 (16.8%)	10 (17.5%)	0.932 (0.361–2.409)	0.885
GG	5 (2.4%)	0 (0.0%)	–––	–––
AG+GG	41 (19.2%)	10 (17.5%)	0.762 (0.301–1.933)	0.568
rs1899663				
GG	137 (64.0%)	39 (68.4%)	1.000 (reference)	
GT	75 (35.0%)	15 (26.3%)	0.724 (0.317–1.651)	0.442
TT	2 (1.0%)	3 (5.3%)	11.131 (1.521–81.490)	0.018 *
GT+TT	77 (36.0%)	18 (31.6%)	0.953 (0.445–2.044)	0.902

DR: diabetic retinopathy, n: number, PDR: proliferative diabetic retinopathy, CI: confidence intervals, AOR: adjusted odds ratio, with their 95% confidence intervals were estimated by multiple logistic regression models after controlling for age, the duration of diabetes, HbA1c, insulin treatment, serum creatinine levels, glomerular filtration rate, and HDL cholesterol levels. * Denotes significant differences between groups.

**Table 6:** Duration of diabetes in diabetic patients according to HOTAIR rs1899663 genotypes.

HOTAIR rs1899663	Duration of Diabetes (years) ^#^	*p* Value
All (n = 728)		
GG (n = 461)	10.74 ± 7.43	0.081
GT+TT (n = 267)	9.74 ± 7.59	
Non-DR (n = 452)		
GG (n = 284)	9.47 ± 6.96	0.766
GT+TT (n = 168)	9.27 ± 7.20	
DR (n = 276)		
GG (n = 177)	12.79 ± 7.73	0.024 *
GT+TT (n = 99)	10.54 ± 8.19	
NPDR (n = 165)		
GG (n = 105)	11.71 ± 6.26	0.096
GT+TT (n = 60)	9.93 ± 7.07	
PDR (n = 111)		
GG (n = 72)	14.35 ± 9.31	0.127
GT+TT (n = 39)	11.46 ± 9.69	

DR: diabetic retinopathy, n: number, NPDR: non-proliferative diabetic retinopathy, PDR: proliferative diabetic retinopathy. # Quantitative data are represented as means ± SD; * denotes significant differences between groups.

## Data Availability

The datasets generated for this study are available upon request to the corresponding authors.

## Supplementary Materials

The following supporting information can be downloaded at: https://www.mdpi.com/article/10.3390/ijerph192114592/s1, Table S1. The context sequences of four HOTAIR SNPs in the study. Figure S1: Representative TaqMan assay for HOTAIR rs920778 genotyping. Figure S2: Representative TaqMan assay for HOTAIR rs12427129 genotyping. Figure S3: Representative TaqMan assay for HOTAIR rs4759314 genotyping. Figure S4: Representative TaqMan assay for HOTAIR rs1899663 genotyping.
