# Supplementary material for: Association of Long Noncoding RNA HOTAIR Polymorphism and the Clinical Manifestations of Diabetic Retinopathy
Source: Int J Environ Res Public Health. 2022 Nov 7;19(21):14592. doi: 10.3390/ijerph192114592 (PMC9658836; doi:10.3390/ijerph192114592)
Supplement: Supplementary file 1 [file ijerph-19-14592-s001.zip › ijerph-1968124-SI.pdf]

Table S1. The context sequences of four HOTAIR SNPs in the study.

Variable	Assay ID	Context Sequence
**HOTAIR (rs920778)**	C___9162435_20	TACAGCTTAAATGTCTGAATGTTAC**[A/G]**GTTTCCTTCAGAAAACAAGGCGGTA
**HOTAIR (rs12427129)**	C___2104247_10	GCCCACTCCGTGGCCAAGGAGCCGG**[C/T]**CAAAGGAGCCGCCCCCAGTAGGTAG
**HOTAIR (rs4759314)**	C__27930754_10	GCTTGGAAGGGATATAAACAGGCCA**[A/G]**GCGGATGCAAGTTAATAAAACCTGA
**HOTAIR (rs1899663)**	C___2104251_20	TCCAAAAGCCTCTAATTGTTGTCAC**[A/C]**TCCACCCTCCTCAACTGGAAAAATG

Figure S1

HOTAIR rs920778

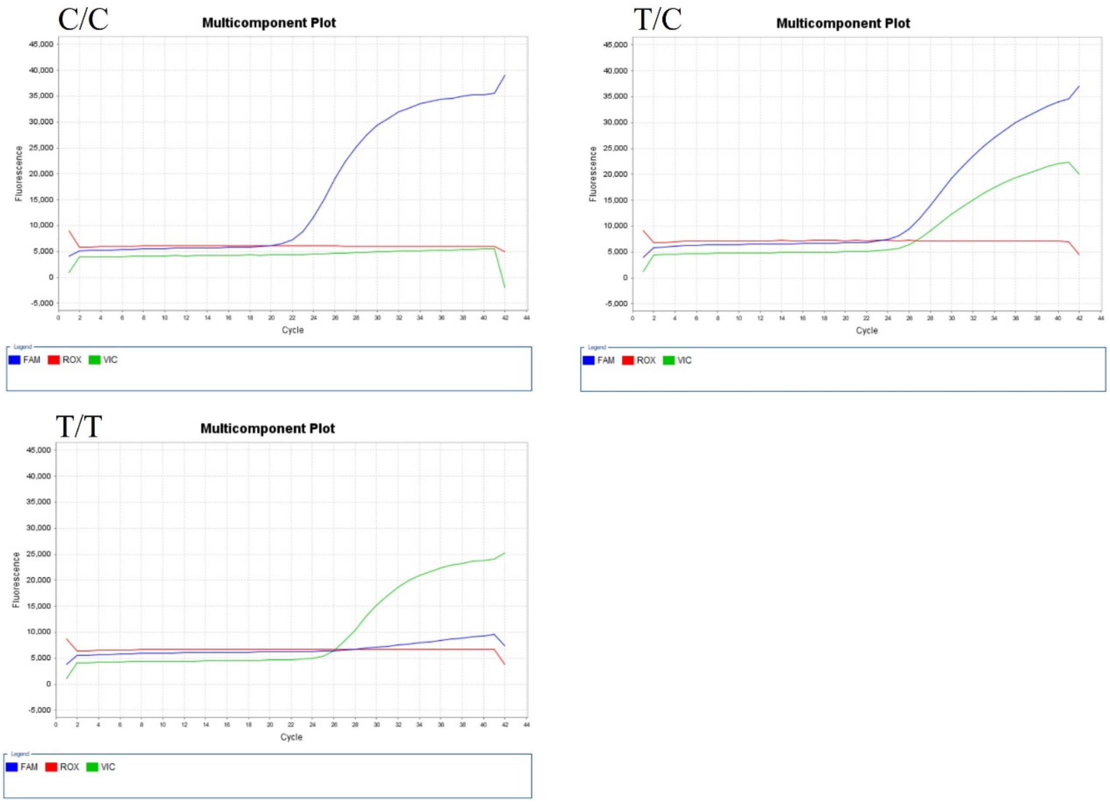

Figure S1: Representative TaqMan assay for HOTAIR rs920778 genotyping.

Figure S2

HOTAIR rs12427129

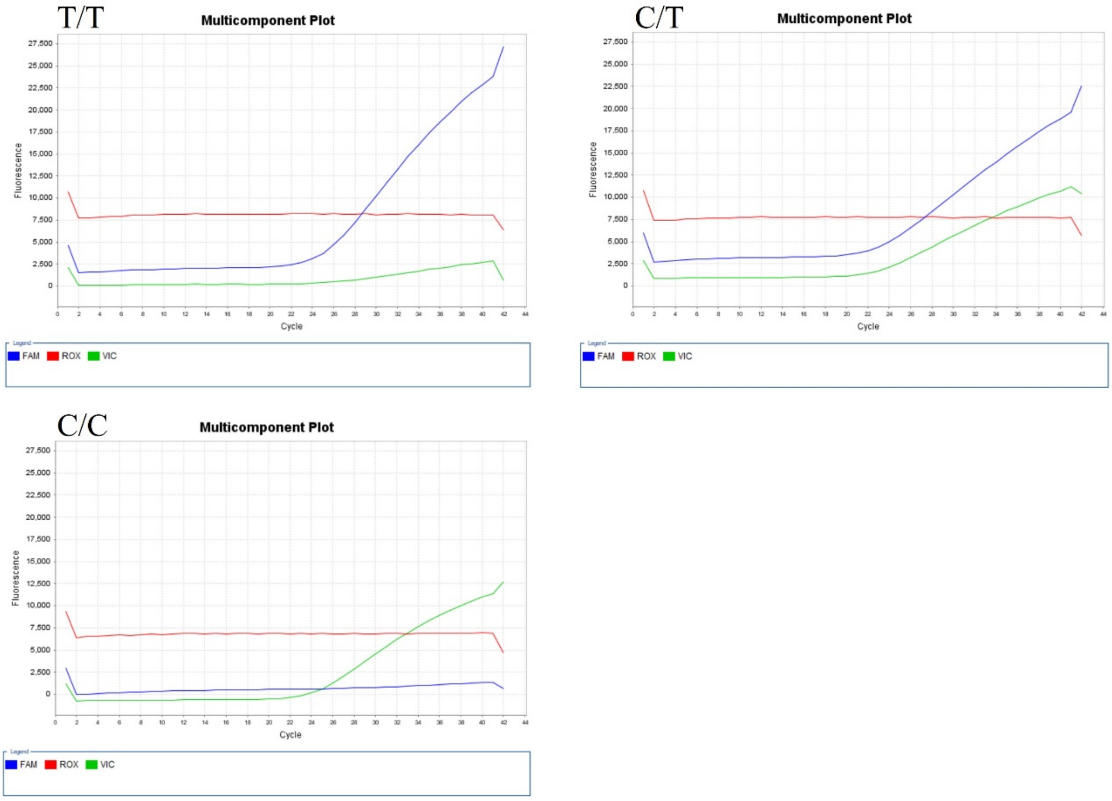

Figure S2: Representative TaqMan assay for HOTAIR rs12427129 genotyping.

Figure S3

HOTAIR rs4759314

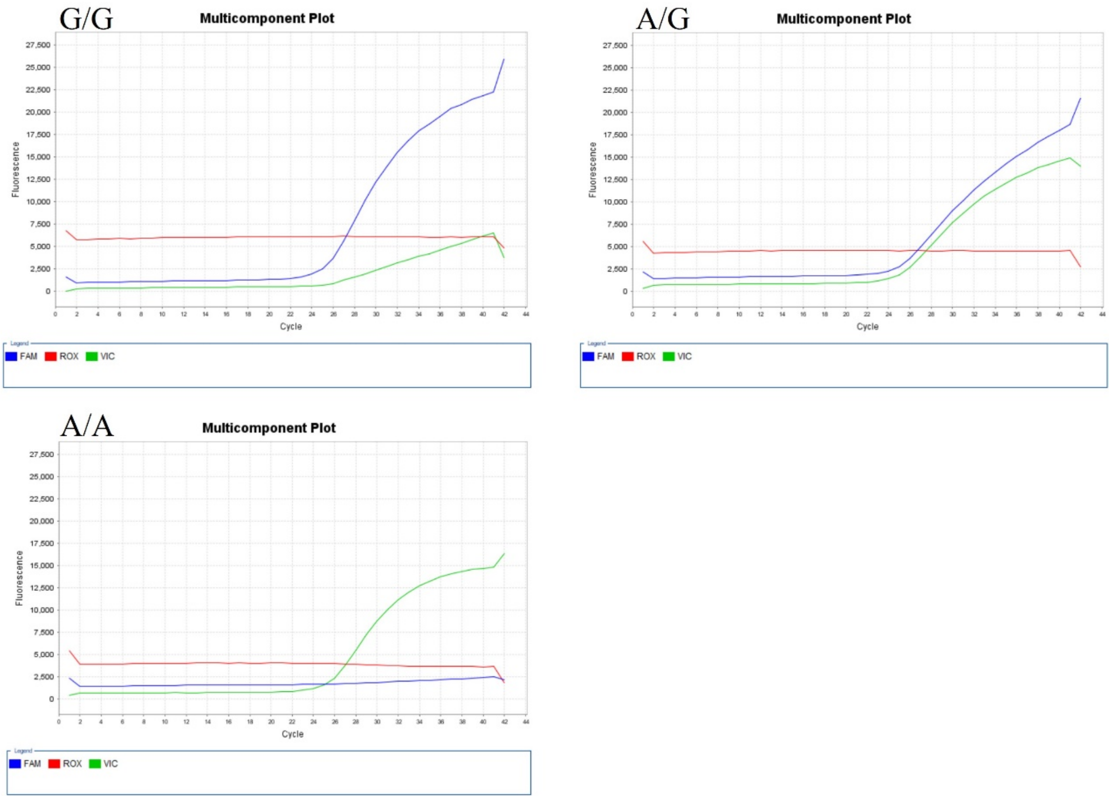

Figure S3: Representative TaqMan assay for HOTAIR rs4759314 genotyping.

Figure S4

HOTAIR rs1899663

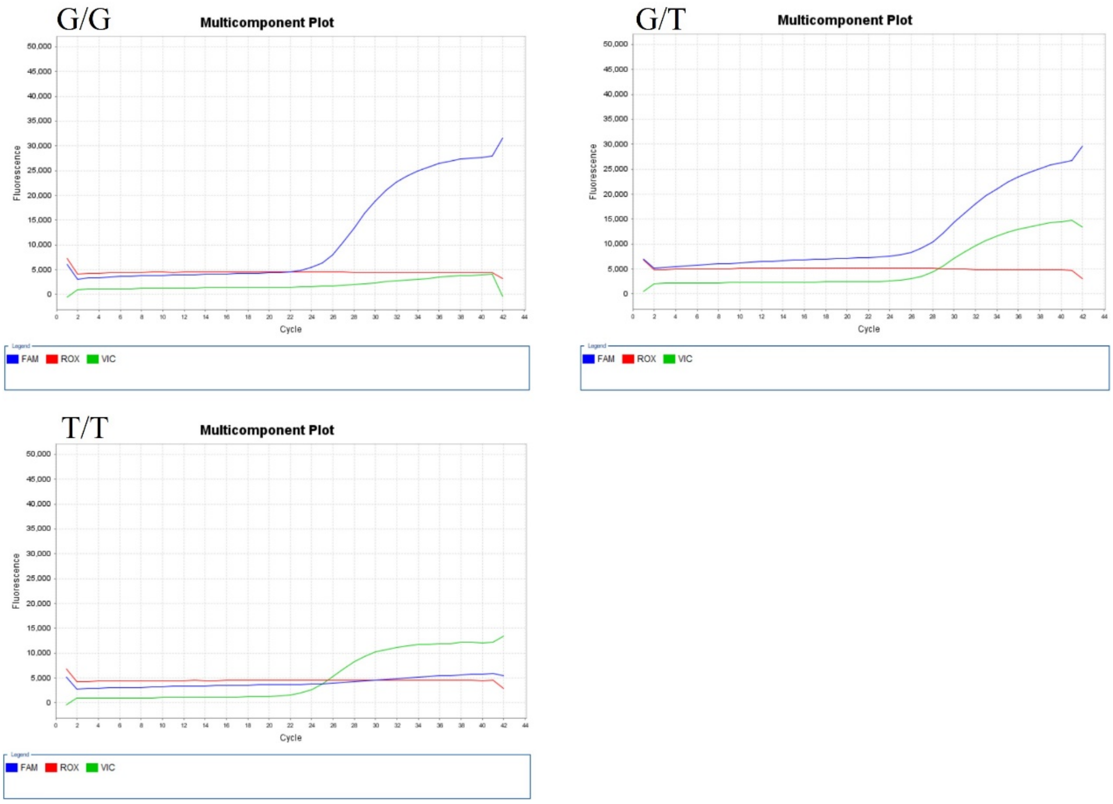

Figure S4: Representative TaqMan assay for HOTAIR rs1899663 genotyping.
